# Comparative analysis of learning motivation, strategies, and effectiveness between medical interns and PGY during the pandemic

**DOI:** 10.1097/MD.0000000000039604

**Published:** 2024-09-13

**Authors:** Chih-Ming Hsu, Shih-Chieh Chuang

**Affiliations:** a Department of Business Administration, School of Management, National Chung Cheng University, Chiayi, Taiwan; b Medical Education Department, Chang Gung Memorial Hospital, Chiayi, Taiwan; c Department of Business Administration, National Central University, Taoyuan, Taiwan.

**Keywords:** COVID-19 pandemic, learning outcomes, medical students, online learning, PGY

## Abstract

In the post-pandemic era, medical education faces significant shifts in learning modes. This study, employing cross-sectional research from 2021 to 2022, surveyed 214 participants, including 104 medical interns and 110 Post-Graduate Year trainees in Taiwan. Findings revealed notable differences between the groups in age and current internship hospital. Medical interns spent significantly more time in daily self-directed learning, with a higher proportion exceeding 3 hours post-work. Although weekly self-directed learning hours did not show statistical significance, more medical students studied over 7 hours weekly. In terms of learning attitudes and motivations, medical interns outscored Post-Graduate Year trainees, indicating a substantial contrast. The study suggests strategic integration of online and traditional education, tailored to course characteristics. Future research should further explore the effectiveness of online learning, aiming to optimize digital learning while preserving traditional education values.

## 1. Introduction

The onset of the COVID-19 pandemic in late 2019 has profoundly impacted various facets of the global healthcare system, including medical education. The conventional mode of medical instruction, once taken for granted, underwent compelled transformations as the severity of the pandemic escalated. The implementation of worldwide preventive measures, such as lockdowns and isolations, further affected the learning experiences of medical students and the methods employed in clinical medical education.

Taking Taiwan as an example, during the peak of the pandemic, the Ministry of Health and Welfare recommended temporarily halting the clinical rotations of medical students or restricting invasive clinical procedures for resident physicians, such as intubations. Consequently, the past 2 to 3 years witnessed significant changes in medical teaching methods. These alterations aimed to minimize interpersonal contact, employing techniques like online distance learning to facilitate uninterrupted education for medical students during the height of the pandemic, mitigating its impact.

Beyond clinical interns, residents in the post-graduate year, responsible for patient care, had to adapt to understanding the infection risks associated with COVID-19 and the changes in clinical care models. In response to the pandemic’s effects on medical education, unconventional approaches like digital and online teaching gradually became the norm. However, these changes come with both advantages and disadvantages. Understanding how to embrace shifts in learning modes in the post-pandemic era and investigating their impacts on medical education is a subject worthy of further exploration. Taiwan sees an annual intake of over 1500 medical students embarking on a 6-year journey of medical education. However, the advent of the COVID-19 pandemic has instigated a transformative shift in traditional teaching methods and assessment approaches.^[1]^ Both the instructional activities in basic and clinical medicine, along with clinical internships, have encountered constraints, leading to reduced learning opportunities and patient care experiences for students. Government-imposed lockdowns and control measures may prohibit students from participating in clinical internships, thereby impacting their training hours. Additionally, students and faculty members face potential infection risks during clinical internships, posing challenges in assessing students’ learning outcomes and necessitating modifications in examination formats.^[2]^

In response to the aforementioned challenges, a novel teaching paradigm has been adopted, with digital learning (Digital learning, E-learning, or online learning) emerging as the norm in education. Digital learning empowers learners to grasp content, sequence, pace, and time, catering to individual learning objectives.^[3]^ Pre-pandemic studies indicate that, compared to traditional learning methods, digital learning shows no significant disparities in the development of behaviors, skills, and knowledge among healthcare professionals, as well as patient outcomes at the clinical front.^[4]^ However, it is noted that digital learning may hold the advantage of enhancing knowledge and skills specifically for medical students, positioning it as a potentially promising instructional approach.^[5]^

In current medical education, both digital learning and traditional teaching prove to be effective learning methodologies. Importantly, the intent is not to replace traditional instructional formats but rather to establish a blended learning approach.^[6]^ Learning motivation encompasses the psychological processes whereby students engage in learning activities, sustain learning behaviors, and achieve objectives.^[7]^ It exerts influence on the direction and emphasis of learning, with students’ self-perception playing a pivotal role in predicting their learning performance. Learning motivation can be categorized into 4 dimensions: value motivation, expectancy motivation, affective motivation, and volitional motivation. Value motivation involves the reasons and beliefs behind students’ engagement in learning activities, expectancy motivation pertains to expectations of learning success, affective motivation concerns emotional responses to learning, and volitional motivation relates to the ability to translate learning into practical action.^[8]^ Learning strategies represent the behavioral and cognitive activities employed by learners throughout the learning process, serving to acquire, retain, and retrieve knowledge. It constitutes a crucial component of metacognitive abilities, wherein learners selectively attend, process, and organize information based on their experiences and learning objectives. This process leads to the internalization of knowledge structures in long-term memory.^[9,10]^ Learning effectiveness is an assessment of the outcomes achieved by students participating in learning activities, measuring changes in certain indicators or behaviors. Its purpose is to enable students to comprehend their learning status, providing teachers with insights for refining instruction and students with a basis for enhancing their learning experiences.^[11]^ Previous research has delved into the relationship between students’ learning motivation, learning strategies, and learning effectiveness in digital learning environments. The findings indicate positive and significant effects in both directions: digital learning motivation influencing learning effectiveness, learning motivation influencing learning strategies, and learning strategies impacting learning effectiveness.

According to research, the majority of students perceive digital or online learning as interactive (77%) and more flexible than traditional teaching methods (58%), allowing them to acquire medical education content. However, a significant portion of students (62%) believes that it cannot fully replace the standard medical education model, and 55% feel that clinical learning cannot be effectively taught through digital means. Another study in Japan explores different online teaching modes, including synchronous online, asynchronous online, and face-to-face instruction. A majority of students (54%) prefer asynchronous online courses, especially among lower-level medical students (basic medical education). The advantages of asynchronous online learning include convenience, reviewability, rewatchability, and the ability to manage one’s schedule. The primary drawback is the inability to adhere to a planned schedule. Clinical clerkship students, compared to basic medical students, prefer synchronous online courses, finding them less stressful for asking questions to instructors. However, the drawback is the inflexibility of course scheduling. Interestingly, a higher proportion of students prefer in-person courses compared to synchronous online, and they also exhibit better academic performance.^[12]^

Currently, the majority of research has been conducted pre-COVID-19, yet the pandemic has induced significant changes in learning modalities. This includes institutional restrictions on medical students’ training in high-risk departments such as infectious diseases and pulmonology. Adjustments in the execution hierarchy of invasive procedures have been made, and traditional meetings have transitioned to online formats. Of greater significance is the post-pandemic era where digital learning, once an exception, has gradually become the norm. In this post-pandemic era, medical students are confronted with adapting to altered learning modes. There is a need to explore the impact of these changes on medical education, particularly the lack of differentiation in the effectiveness of online learning during and before the pandemic in the field of medical education. Consequently, this study aims to compare the relationships between medical students and Post-Graduate Year (PGY) in terms of learning motivation, strategies, and effectiveness. The goal is to provide pertinent recommendations and reform directions for future medical education models.

## 2. Method

### 2.1. Research context and participants

In a cross-sectional research design, purposive sampling was employed to conduct a study from 2021 to 2022 across various teaching hospitals in Taiwan. The participants included PGY trainees who had completed 2 years of training after graduating and medical students involved in clinical internships. The investigation was carried out using an online survey methodology. Ethical approval for this study was obtained from Chang Gung Medical Foundation Institutional Review Board.

### 2.2. Research instrument

The questionnaire utilized in this study was a self-designed structured questionnaire, comprising 2 main sections: (1) basic information and (2) a scale measuring learning motivation, attitudes, and behaviors towards the use of online educational resources.

Firstly, the analysis of “Learning Behavior” primarily aims to understand participants’ usage of online materials for learning, comprising a total of 4 questions. Secondly, the section on “Perception of Online Learning” draws inspiration from the work of Bączek et al, specifically “Students’ perception of online learning during the COVID-19 pandemic.” This section focuses on gaining insights into the perspectives of Taiwanese medical students regarding online learning, consisting of 15 questions, including 9 questions describing the advantages of online learning. The third part, the “Learning Attitude” scale, is adapted from the questionnaire developed by Professor Wu Mingda in the “Gaozhan Project Student Learning Attitude Questionnaire.” It explores learning attitudes across 3 dimensions: “Attitude towards the online course” (α = 0.94), “Attitude towards the teacher” (α = 0.88), and “Attitude towards classmates” (α = 0.93). This part primarily aims to understand the impact of online courses, instructors, and classmates on the attitudes of medical students towards using online learning, totaling 21 questions. Finally, the “Learning Motivation” scale for online learning is based on Tuan and Shieh “Student Motivation Toward Science Learning” (α = 0.89), focusing on understanding the motivation of medical students using online courses, comprising a total of 35 questions, including 4 designed as negative statements.

The questionnaire content was collaboratively developed by the research team, drawing upon relevant literature. It underwent expert validation to ensure its appropriateness for the study. The design employed a structured questionnaire method and Likert scales, with Likert scale points ranging from 1 (strongly disagree) to 5 (strongly agree). Three experts in the relevant field were commissioned for expert content validity assessment using the Content Validity Index scoring method. The Content Validity Index was employed to assess the consistency of expert opinions regarding the questionnaire’s content. After removing non-significant items, the researcher, guided by expert suggestions, made appropriate modifications to the wording and phrasing of the questionnaire. Each section of the questionnaire achieved a content validity score of 0.90 or above, indicating consensus among experts. Subsequently, the finalized questionnaire was prepared for formal use.

### 2.3. Data collection

This study utilized the free software SurveyCake for data collection. Participant recruitment strategies included: (1) utilizing a Facebook group, comprising prospective interns about to enter PGY training, alongside physicians who are currently undergoing or have completed PGY training. The recruitment information was disseminated within the group by a co-host who is a PGY trainee. (2) Invitation through online courses: participants in these courses consisted of interns and PGY trainees from various hospitals. Recruitment efforts were conducted during the pre-course and post-course breaks to optimize participation.

### 2.4. Data analysis

Following the collection of survey responses, statistical analysis was conducted using SPSS version 20.0. The obtained statistical results were then utilized for in-depth research analysis and inference.

## 3. Result

This study enrolled a total of 214 participants, comprising 104 medical interns and 110 PGY individuals. Background homogeneity tests were conducted on medical interns and PGY participants using the chi-square test and Fisher exact test. The results, as presented in Table 1, revealed significant differences in age (*P* < .01) and the current hospital of internship (*P* < .01). This indicates a notable variation in the proportions of interviewed medical interns and PGY participants concerning different age groups and the hospitals where they are currently undergoing internship. However, no significant differences were observed in the remaining background variables.

**Table 1:** Homogeneity test results of group and background variables.

Item	Total (N = 214)	Group		χ^2^/*P*
		Medical interns (n = 104)	PGY (n = 110)	
Age				20.90/<.01**
20–25	142 (66.4%)	54 (51.9%)	88 (80.0%)	
26–30	67 (31.3%)	47 (45.2%)	20 (18.2%)	
31–35	4 (1.9%)	3 (2.9%)	1 (0.9%)	
36–40	1 (0.4%)	0 (0.0%)	1 (0.9%)	
Gender				0.04/0.84
Male	90 (42.1%)	43 (41.3%)	47 (42.7%)	
Female	124 (57.9%)	61 (58.7%)	63 (57.3%)	
Graduated from				1.09/0.30
Public university	91 (42.5%)	48 (46.2%)	43 (39.1%)	
Private university	123 (57.5%)	56 (53.8%)	67 (60.9%)	
Internship at				6.75/<.01**
Medical center	74 (34.6%)	45 (43.3%)	29 (26.4%)	
Regional hospitals	140 (65.4%)	59 (56.7%)	81 (73.6%)	
Graduation grade				0.06/1.00
Top 25%	14 (6.5%)	7 (6.7%)	7 (6.4%)	
26–50%	48 (22.4%)	23 (22.1%)	25 (22.7%)	
51–75%	69 (32.2%)	33 (31.7%)	36 (32.7%)	
76–100%	83 (38.8%)	41 (39.4%)	42 (38.2%)	
Specialty preference of resident Doctor				0.55/0.93
Internal Medicine	49 (22.9%)	22 (21.2%)	27 (24.5%)	
General surgery	39 (18.2%)	19 (18.3%)	20 (18.2%)	
Obstetrics and Gynecology/Pediatrics	9 (4.2%)	4 (3.8%)	5 (4.5%)	
Others	117 (54.7%)	59 (56.7%)	58 (52.7%)	

** *P* < .01.

Based on Table 2, significant differences were observed in the average daily self-directed learning time after work over the past 3 months between medical interns and PGY trainees. The average daily self-directed learning time (*P* < .01) was notably different, with a higher proportion of medical interns spending over 3 hours in self-directed learning after work. However, there was no significant difference in the average non-working hours.

**Table 2:** Group and homogeneity test results of “Learning Behavior”.

Item	Total (N = 214)	Group		χ^2^/*P*
		Medical interns (n = 104)	PGY (n = 110)	
Learning time after work per day past 3 months				17.09/<.01**
3 hours and above	54 (25.2%)	36 (34.6%)	18 (16.4%)	
1–3 hours	124 (57.9%)	60 (57.7%)	64 (58.2%)	
Less than 1 hour	36 (16.8%)	8 (7.7%)	28 (25.5%)	
Learning time during non-working hours per week past 3 months				7.19/.07
7 hours or more	73 (34.1%)	44 (42.3%)	29 (26.4%)	
3–7 hours	81 (37.9%)	37 (35.6%)	44 (40.0%)	
1–3 hours	37 (17.3%)	13 (12.5%)	24 (21.8%)	
Less than 1 hour	23 (10.7%)	10 (9.6%)	13 (11.8%)	

** *P* < .01.

According to the Table 2, it can be observed that in terms of daily self-directed learning time, over half of the PGY trainees and medical students dedicate between 1 to 3 hours to learning each day. However, the proportion of individuals spending more than 3 hours in learning is significantly higher among medical students compared to PGY trainees. Regarding the weekly self-directed learning hours during non-working hours, although the statistical difference is not significant, among the groups studying for 7 hours or more per week, the proportion of medical students remains higher. This suggests that there may be a considerable correlation between self-directed learning time and the content of daytime work and study. The job tasks of intern medical students are primarily oriented toward “learning” as they are still in a student role. Once they finish work on time, the clinical internship duties conclude, allowing them more time for self-directed learning. Additionally, medical students must prepare for the national physician specialty examination after graduation. The additional pressure may drive them to invest more time in self-directed learning to ensure excellent academic performance. In contrast, PGY trainees are already hospital staff, primarily engaged in “clinical work,” including patient care and practical clinical internships. Furthermore, PGY trainees have other responsibilities such as writing papers and participating in morning meeting presentations. Due to the nature of their work, PGY trainees cannot completely separate from clinical tasks even after working hours. Consequently, the self-directed learning time for PGY trainees is significantly less. Moreover, PGY trainees may have professional development needs, requiring continuous advancement in their careers, which could involve participating in training courses, research, academic conferences, and more. These demands may occupy their time, making it challenging for them to engage in additional self-directed learning.

According to Table 3, the overall average for learning attitudes is 3.54, with a standard deviation of 0.63. The average learning attitude for internship medical students is 3.68, with a standard deviation of 0.61. For PGY trainees, the average learning attitude is 3.42, with a standard deviation of 0.63. These values indicate a tendency towards agreement with the learning attitude statements.

**Table 3:** Descriptive statistics of learning attitudes and motivation.

Scale and dimensions	Mean (SD)		
	Total	Medical interns	PGY
Learning attitude	3.54 (0.63)	3.68 (0.61)	3.42 (0.63)
Course	3.47 (0.80)	3.66 (0.74)	3.28 (0.82)
Teacher	3.78 (0.60)	3.84 (0.59)	3.72 (0.61)
Classmates	3.37 (0.73)	3.51 (0.70)	3.24 (0.74)
Learning motivation	3.63 (0.48)	3.74 (0.43)	3.53 (0.50)
Self-efficacy	3.30 (0.58)	3.30 (0.53)	3.30 (0.63)
Proactive learning strategies	3.99 (0.63)	4.10 (0.61)	3.88 (0.63)
Learning value	3.97 (0.67)	4.14 (0.66)	3.81 (0.64)
Performance goals	2.95 (0.89)	3.06 (0.96)	2.84 (0.81)
Achievement goals	3.78 (0.67)	3.84 (0.66)	3.72 (0.67)
Learning environmental incentives	3.61 (0.62)	3.77 (0.57)	3.46 (0.64)

Additionally, motivation questions 2, 4, 5, and 6 were reverse-coded, and the overall average for learning motivation is 3.63, with a standard deviation of 0.48. The average learning motivation for internship medical students is 3.74, with a standard deviation of 0.43. For PGY trainees, the average learning motivation is 3.53, with a standard deviation of 0.50. These values indicate a positive inclination towards the learning motivation statements.

Based on Table 4, it is evident that the groups significantly differ in the subdomains of learning attitude (t = 3.09(212), *P* < .01), including course (t = 3.54 (212), *P* < .01) and classmates (t = 2.70(212), *P* < .01), with medical interns scoring significantly higher than PGY. In terms of learning motivation, the groups exhibit significant differences in motivation (t = 3.21(212), *P* < .01) and its subdomains, including proactive learning strategies (t = 2.56(212), *P* < .05), learning value (t = 3.75 (212), *P* < .01), and learning environmental incentives (t = 3.66 (212), *P* < .01). Once again, internship medical students score significantly higher than PGY in these aspects.

**Table 4:** Differences in learning attitudes and motivation between medical interns and Post-Graduate Year trainees.

Scale and Dimensions	Group	Mean	SD	t	*P*
Learning	Medical interns	3.68	0.61	3.09**	<.01
Attitude	PGY	3.42	0.63		
Course	Medical interns	3.66	0.74	3.54**	<.01
	PGY	3.28	0.82		
Teacher	Medical interns	3.84	0.59	1.52	0.13
	PGY	3.72	0.61		
Classmates	Medical interns	3.51	0.70	2.70**	<.01
	PGY	3.24	0.74		
Learning	Medical interns	3.74	0.43	3.21**	<.01
Motivation	PGY	3.53	0.50		
Self-Efficacy	Medical interns	3.30	0.53	−0.06	0.95
	PGY	3.30	0.63		
Proactive	Medical interns	4.10	0.61	2.56*	0.01
Learning Strategies	PGY	3.88	0.63		
Learning value	Medical interns	4.14	0.66	3.75**	<.01
	PGY	3.80	0.64		
Performance	Medical interns	3.06	0.96	1.85	0.07
Goals	PGY	2.84	0.81		
Achievement	Medical interns	3.84	0.66	1.39	0.17
Goals	PGY	3.72	0.67		
Learning	Medical interns	3.77	0.57	3.66**	<.01
Environmental incentives	PGY	3.46	0.64		

* *P* < .05.

** *P* < .01.

## 4. Discussion

During the pandemic, digital learning has transitioned from an exception to a norm, and post-pandemic, medical students in their internship years are required to adapt to changes in the learning environment. This study offers preliminary insights into the relationship between the learning motivations, strategies, and outcomes of internship medical students and PGY. Examining the attitudes, strategies, and outcomes of learning during the pandemic, the research compares the differences in online learning between internship medical students and PGY. The findings suggest that, overall, internship medical students exhibit more positive attitudes and higher motivation towards online classes compared to PGY.

In pre-pandemic research, digital learning was considered to have relatively little difference in cultivating knowledge and skills among medical professionals, but it demonstrated potential in enhancing the knowledge and skills of medical interns.^[4,5]^ However, this study also reveals that digital learning cannot entirely replace traditional medical education models, especially in the realm of clinical learning. Students generally acknowledge the interactivity and flexibility of digital learning but maintain the belief that it cannot fully substitute traditional teaching methods.

The motivation to learn is crucial for students to engage in learning activities, sustain learning behavior, and achieve goals. Students’ self-perception plays a pivotal role in the learning process, influencing their learning direction and focus.^[13]^ Additionally, the value assigned to learning and the employed learning strategies constitute vital components of students’ learning experiences, aiding in the acquisition, retention, and retrieval of knowledge. These findings underscore the importance of fostering students’ learning motivation and strategies, especially in digital learning environments.

The research findings reveal that the overall average score for learning attitudes is relatively high, indicating a general agreement among students on attitude-related items. However, the average learning attitude score for medical interns is slightly higher than that of PGY, suggesting a greater enthusiasm for learning motivation among medical interns. On the other hand, the overall average score for learning motivation is also high, indicating a positive inclination among students towards motivational aspects. Nevertheless, the learning motivation average score for medical interns is significantly higher than that of PGY, indicating a heightened motivation among them in engaging in learning activities, sustaining learning behavior, and achieving goals.

Intern medical students may have higher learning motivation than PGY for various reasons. Firstly, they likely face greater academic pressure. Interns typically find themselves in the final stages of their medical curriculum, preparing for crucial academic assessments such as the national medical specialty exams. These exams are pivotal for their future career development, leading to heightened academic stress. Striving for excellence, interns may actively dedicate more time and effort to self-directed learning. Secondly, the need for career development could serve as a powerful motivator for intern medical students. Positioned in the early stages of their medical careers, they may prioritize their professional growth and future medical trajectory. Seeking to accumulate more experience during clinical internships, they aim to establish a strong professional foundation. This desire for career development may inspire them to engage more actively in self-directed learning, enhancing their clinical skills and knowledge. Additionally, interns might be passionate about clinical practice, being in a phase of learning and applying medical knowledge. This enthusiasm could drive them to invest additional time in in-depth case studies, participation in discussions, and reading relevant literature, expanding their clinical perspectives.

Xiaonan Hao et al,^[14]^ in a systemic review, examined the application of digital education for nursing and medical interns during the COVID-19 pandemic. The analysis synthesized recent evidence on the effectiveness of digital education modalities, including virtual reality-based simulation training, teleconsultation, web-based specialized skills learning, and multimodal online curriculums. Regarding virtual reality-based simulation training, the review found that it can be a valuable alternative to bedside clinical practice, with studies reporting positive impacts on interns’ knowledge, confidence, and overall performance.^[15–17]^ Similarly, teleconsultation and virtual rounds were shown to maintain the continuity of clinical practicum, providing interns with valuable experiences and interactions with patients and clinicians.^[18–20]^ Web-based specialized skills learning, such as online surgical skills learning and virtual radiology review, proved to be effective in enhancing interns’ learning experiences and engagement.^[21,22]^ Additionally, multimodal online curriculums, including synchronous and asynchronous content, were found to be well-received by interns, particularly when they involved interactive discussion learning patterns.^[23,24]^ The review also highlighted the importance of considering learning group size and teaching resources in digital education. Smaller learning group sizes were found to be more effective for clinical teaching, allowing for deeper exploration and greater participation.^[25]^ Furthermore, interactive training was preferred over passive access to resources, indicating the importance of engaging teaching methods in digital education.^[26,27]^

In a multicenter study, Samiullah Dost et al^[28]^ investigated the perceptions of 2721 UK medical students from 39 medical schools towards online teaching during the COVID-19 pandemic. The study found a significant increase in the time spent by medical students on online teaching platforms. Video tutorials were perceived as the most effective, followed by online question banks and live tutorials. Clinical students found live tutorials to be the most effective, while preclinical students favored video tutorials. However, the high flow of resources has led to choice overload, potentially increasing burnout rates among students. Students expressed a desire for more interactive online teaching sessions, which could be achieved through the incorporation of student response systems such as polls, quizzes, breakout rooms, and online QA sessions. Previous literature suggests that the incorporation of these tools was able to improve student engagement.^[29–32]^ However, students may express concerns about the quality of online resources produced during the pandemic and their impact on clinical competence.^[33]^

Based on the research findings, our study provides the following recommendations. Firstly, considering the highly specialized nature of medical training, not all courses are suitable for online delivery. It is suggested that medical schools categorize courses for students, identifying those conducive to online formats, encompassing both synchronous and asynchronous components. This categorization should guide how clinical teaching is conducted online. Moreover, for hands-on clinical skill subjects, incorporating partial online courses may serve as effective tools for pre-learning and post-review purposes. Secondly, tailored online courses should be developed for intern medical students and PGY to address their distinct needs, optimizing the learning outcomes of online education for both groups. Future research is warranted to delve deeper into the differences between online learning effectiveness during the pandemic and pre-pandemic learning outcomes. This includes exploring how to maximize the potential of digital learning while preserving the traditional educational values.

## 5. Limitation

This study was conducted during the COVID-19 pandemic, and the findings may not be generalizable to all learning contexts or countries, representing a significant limitation of this research.

## 6. Conclusion

During the pandemic, there has been a shift in the learning modes of medical interns and PGY trainees, with the widespread adoption of digital learning. Research findings indicate that the attitude and motivation of medical interns towards online learning are more positive compared to PGY trainees. This difference may be attributed to the higher academic pressure and stronger career development needs experienced by medical interns. Digital learning offers advantages such as interactivity and flexibility in time and location, yet it cannot fully replace traditional teaching, especially in clinical learning. The motivation to learn is crucial for students, influencing their engagement in learning and goal attainment. In conclusion, this study recommends grouping medical curriculum, selecting subjects suitable for online learning, and providing online previews and reviews for clinical skill subjects. Future research should delve into the effectiveness of online learning during the pandemic, maximizing the potential of digital learning while preserving the values of traditional teaching.

## Author contributions

**Conceptualization:** Chih-Ming Hsu, Shih-Chieh Chuang.

**Data curation:** Chih-Ming Hsu.

**Formal analysis:** Chih-Ming Hsu.

**Funding acquisition:** Chih-Ming Hsu.

**Investigation:** Chih-Ming Hsu.

**Methodology:** Chih-Ming Hsu, Shih-Chieh Chuang.

**Project administration:** Chih-Ming Hsu.

**Resources:** Chih-Ming Hsu.

**Software:** Chih-Ming Hsu, Shih-Chieh Chuang.

**Supervision:** Chih-Ming Hsu, Shih-Chieh Chuang.

**Validation:** Chih-Ming Hsu.

**Visualization:** Chih-Ming Hsu.

**Writing – original draft:** Chih-Ming Hsu.

**Writing – review & editing:** Chih-Ming Hsu, Shih-Chieh Chuang.
